# Magnetic Resonance Imaging Features in Intramedullary Tumors: A Pictorial Review

**DOI:** 10.3390/biomedicines14061239

**Published:** 2026-05-29

**Authors:** Corentin Dauleac, David Meyronet, François Ducray, Patrick Mertens, François Cotton

**Affiliations:** 1 Spinal Cord and Peripheral Nerve Neurosurgery Department, Hospices Civils de Lyon, Hôpital Neurologique et Neurochirurgical Pierre Wertheimer, 69500 Lyon, France; patrick.mertens@chu-lyon.fr; 2 Laboratoire CREATIS, INSA-Lyon, Université Lyon I, Université Claude Bernard, 69621 Lyon, France; francois.cotton@chu-lyon.fr; 3 Department of Neuro-Pathology, Hospices Civils de Lyon, Hôpital Neurologique et Neurochirurgical Pierre Wertheimer, 69500 Lyon, France; david.meyronet@chu-lyon.fr; 4 Department of Neuro-Oncology, Hospices Civils de Lyon, Hôpital Neurologique et Neurochirurgical Pierre Wertheimer, 69500 Lyon, France; francois.ducray@chu-lyon.fr; 5 Department of Radiology, Hospices Civils de Lyon, Hôpital Lyon Sud, 69495 Lyon, France

**Keywords:** spinal cord, intramedullary tumor, MRI, DTI

## Abstract

**Background/Objectives**: Intramedullary tumors are uncommon spinal cord lesions that account for a small proportion of central nervous system neoplasms but are associated with a high risk of neurological morbidity. Accurate preoperative characterization is essential because therapeutic strategies, surgical planning, and functional prognosis depend strongly on tumor biology and growth behavior within the confined spinal cord environment. This study aims to characterize the radiological phenotype of intramedullary tumors and to identify imaging patterns that may assist in lesion characterization and diagnostic stratification. **Methods**: A retrospective analysis of preoperative MRI findings in patients with histopathologically confirmed intramedullary tumors was performed. Preoperative MRI examinations were systematically analyzed to describe imaging features according to tumor histology using conventional sequences (T1-weighted, T2-weighted, and contrast-enhanced imaging). **Results**: Distinct radiological phenotypes were observed across a wide spectrum of lesions. Glial tumors, including subependymoma, ependymoma, pilocytic astrocytoma, diffuse midline glioma H3K27M, glioblastoma, high-grade astrocytoma with piloid features, ganglioglioma, and diffuse leptomeningeal glioneural tumors, demonstrated variable combinations of cord expansion, margin definition, enhancement patterns, and tract involvement, reflecting differences between expansile and infiltrative growth. Secondary tumors such as metastases frequently exhibited aggressive imaging features, including extensive edema and intense or heterogeneous enhancement. Vascular lesions, including hemangioblastoma and cavernoma, showed characteristic vascular signatures, such as nodular enhancement with flow voids or susceptibility-related signal changes. Developmental lesions, such as epidermoid cysts, neurenteric cysts, and lipoma, displayed distinctive signal characteristics, especially on diffusion and T1, that aided differentiation from neoplastic processes. **Conclusions**: In conclusion, the structured radiological interpretation functions proposed herein are not only useful for diagnostic purposes, but could also be useful for risk stratification and therapeutic guidance.

## 1. Introduction

Intramedullary tumors represent a heterogeneous group of lesions that originate within the spinal cord parenchyma, accounting for approximately 2–4% of all central nervous system (CNS) neoplasms and 30–47% of intradural tumors in adults [1]. Despite their rarity, these lesions are of major clinical importance because of the high risk of neurological sequelae and the challenges inherent in their diagnosis and surgical management. The compact anatomy of the spinal cord makes accurate preoperative characterization essential [2,3], as treatment strategies depend heavily on the underlying histological and molecular nature of the tumor.

Magnetic resonance imaging (MRI) is the cornerstone of spinal cord tumor evaluation. In recent years, advances in MRI technology have improved the radiological approach to spinal cord tumors [4,5]. It offers unparalleled soft-tissue contrast and the ability to define tumor morphology, cord expansion, cystic components, and patterns of enhancement [5]. MRI not only serves diagnostic purposes but also plays a crucial role in surgical planning [6], assessment of resectability [7,8], and postoperative follow-up. Moreover, multiparametric imaging may provide new insights into the tumor microstructure of the spinal cord [9,10,11], potentially improving lesion characterization beyond conventional morphology.

In parallel, the 2021 World Health Organization (WHO) classification of CNS tumors introduced integrated molecular diagnostics incorporating genetic and epigenetic alterations into histopathological definitions [12,13]. This paradigm shift has established a biologically driven framework in which imaging is increasingly expected to mirror tumor behavior rather than merely depict anatomy. However, in the spinal cord, radiological knowledge originates from small, heterogeneous series, and rare tumor entities remain poorly described or are extrapolated from intracranial counterparts despite distinct spinal microenvironmental constraints.

The aim of this review is therefore to provide a comprehensive and structured overview of the neuroimaging features of intramedullary tumors, organized according to the main histological entities encountered in clinical practice (Table 1). To achieve this, we retrospectively reviewed preoperative spinal cord MRIs in patients with histopathologically confirmed intramedullary tumors in order to systematically illustrate and describe their imaging patterns. This approach seeks to support clinicians in the differential diagnosis of intramedullary lesions and to promote a radiological framework aligned with the current WHO classification paradigm.

## 2. Methodology and MRI Protocol

All procedures performed in this study involving human participants were approved by the Ethics Committee (IRB 00011687–2026/26) on 26 April 2026 and were conducted in accordance with the 1964 Declaration of Helsinki and its later amendments or comparable ethical standards.

All cases of intramedullary tumor included in this retrospective study had histopathological confirmation following surgical resection or biopsy, in accordance with the current WHO classification criteria. This study was not designed as a cohort-based analysis with consecutive patient inclusion, but rather as an illustrative review based on representative, histopathologically confirmed cases selected to reflect typical imaging patters of each tumor entity. Only patients with available preoperative MRI examinations of sufficient quality were included. For each tumor entity, a single representative case was selected to illustrate its characteristic imaging features. MRI studies were reviewed to assess conventional imaging features, including lesion location, longitudinal extent, signal characteristics, and contrast enhancement patterns, with additional evaluation of advanced imaging techniques when available. MRI examinations were interpreted by a senior radiologist and systematically reviewed by a neuroradiologist with more than 30 years of experience.

Given the complex microanatomy and the small cross-sectional area of the spinal cord, high-resolution imaging protocols are indispensable for accurate lesion characterization. The principal objectives of MRI evaluation are to determine the lesion’s longitudinal and axial extent, its relationship with the spinal cord parenchyma, its internal architecture, and its enhancement pattern.

### 2.1. Technical Considerations

High-field MRI scanners (3 Tesla) are recommended for improved signal-to-noise ratio and spatial resolution. Thin-slice acquisitions (≤2 mm) are preferred to minimize partial volume effects. Sagittal imaging is optimal for assessing the craniocaudal extent, whereas axial imaging provides essential details about tumor localization within the cord, infiltration, and peritumoral changes. The use of dedicated spine coils or multi-channel phased-array coils enhances image quality.

A standard MRI examination should include sagittal and axial T1-weighted, T2-weighted, as well as post-gadolinium T1-weighted imaging with a 3–5-min delay post-injection to allow optimal contrast uptake. Coronal MRI sequences have limited utility in the evaluation of strictly intramedullary spinal cord tumors since the cranio-caudal extent of lesions is optimally assessed on sagittal images, while axial sequences provide detailed information regarding lesion symmetry, eccentricity, and relationship to spinal cord structures (Table 2).

### 2.2. Sagittal View

The sagittal view is the most informative for assessing the craniocaudal extent of intramedullary tumors and their relationship to the spinal cord. Because intramedullary tumors typically expand the spinal cord rather than displace it, careful evaluation of the degree of swelling and signal extension beyond the visible mass is essential. Lesions are conventionally categorized as follows: (1) focal (<2 vertebral segments; e.g., hemangioblastomas, metastases…), (2) intermediate (2–3 segments; e.g., typical of low-grade astrocytomas or gangliogliomas), and (3) longitudinally extensive (>3 segments, e.g., diffuse astrocytomas, H3K27-altered gliomas).

### 2.3. Axial View

Axial images provide a cross-sectional understanding of tumor location relative to the central canal, gray matter, and white matter tracts, and are keys for evaluating tract infiltration. Central lesions are usually represented by ependymomas, which originate from the ependymal lining and symmetrically expand the cord. Eccentric lesions are characteristic of astrocytomas and gangliogliomas, which arise from glial cells within the hemicord, producing asymmetric expansion. Subpial or exophytic lesions, including hemangioblastomas, cavernomas, and metastases, are located near the cord surface and may exhibit pial-vessel involvement or exophytic components extending into the subarachnoid space.

### 2.4. Enhancement Patterns: Morphological and Diagnostic Implications

Gadolinium-enhanced MRI is crucial for evaluating blood–spinal cord barrier integrity and remains one of the most powerful differentiating tools among intramedullary lesions. The pattern, intensity, and distribution of enhancement offer valuable insights. Homogeneous, intense enhancement throughout the lesion is typical of ependymomas and lymphomas. According to the type of ependymoma (cystic contingent), enhancement may be more heterogeneous due to an intra-tumoral cystic component, whereas lymphomas enhance homogeneously despite low perfusion due to high cellularity. Heterogeneous or ring-like enhancement is characteristic of astrocytomas, particularly high-grade variants and glioblastomas, which are caused by necrosis and heterogeneous vascular permeability. Nodular enhancement, frequently associated with pial-flow voids or feeding vessels, is characteristic of hemangioblastomas. Minimal or absent enhancement may be seen in some low-grade tumors, including pilocytic astrocytomas and oligodendrogliomas. The absence of enhancement should not exclude neoplasia, as it may simply reflect an intact blood–spinal cord barrier.

## 3. Radiological Features of Intramedullary Tumors

### 3.1. Primary CNS Tumors

#### 3.1.1. Spinal Ependymal Tumors

##### Subependymoma (Figure 1A)

Spinal subependymomas most commonly involve the cervical and upper thoracic spinal cord and are typically located in a central or slightly eccentric intramedullary position, often in close relationship to the central canal [14]. They present as well-circumscribed, non-infiltrative masses. On MRI, they are generally iso- to hypointense on T1-weighted sequences and hyperintense on T2-weighted images, often with a remarkably homogeneous signals [15]. Peritumoral edema is minimal or absent, reflecting the non-invasive nature of the lesion. A marked fusiform spinal cord enlargement with abrupt margins on sagittal MRI creates the characteristic “bamboo leaf sign,” reflecting subpial tumor growth [16]. Gadolinium enhancement is absent or minimal in most cases. When present, enhancement is usually faint. The lack of intense or homogeneous enhancement is a key feature differentiating subependymomas from classic ependymomas [14,15].

**Figure 1:**
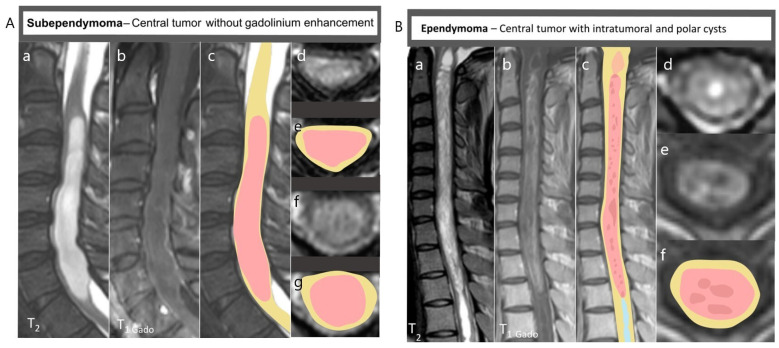
(**A**) **Subependymoma:** (**a**) Sagittal T2-weighted image showing an extensive well-defined hypersignal-T2 mass within the cord; (**b**) Sagittal T1-contrast-enhanced image showing the absence of gadolinium enhancement; (**c**) Sagittal pictorial representation; (**d**,**f**) Axial T2-weighted image showing the central tumor; (**e**,**g**) Axial pictorial representation. (**B**) **Ependymoma:** (**a**) Sagittal T2-weighted image showing an extensive well-defined mass with multiple intumoral cysts and polar cyst; (**b**) Sagittal T1-contrast-enhanced image showing the gadolinium enhancement of the tumor associated with the polar cyst; (**c**) Sagittal pictorial representation; (**d**) Axial T2-weighted image showing a central tumor with an intratumoral cyst; (**e**) Axial T1-gadolinium enhanced image showing gadolinium enhancement of the tumor without enhancement of the cysts; (**f**) Axial pictorial representation.

##### Spinal Ependymoma (Figure 1B)

Ependymomas are the most frequent intramedullary tumor in adults, accounting for approximately 60% of adult cases and 30% of those in children. Arising from the ependymal lining of the central canal, these tumors are typically centrally located, symmetrical, well circumscribed, and occur most frequently in the cervical or cervicothoracic region [17]. They often span several vertebral segments and may be associated with both polar cysts and syringomyelia, the latter resulting from altered cerebrospinal fluid (CSF) dynamics. Ependymomas are typically iso- to hypointense on T1, with a sharp demarcation from the surrounding spinal cord [18]. A highly characteristic feature is the hemosiderin “cap sign”, a T2/SWI hypointense rim at one or both poles of the tumor caused by chronic microhemorrhage (Table 2). Internal signal heterogeneity may arise from hemorrhage or cystic components (which were present in more than 60% of ependymomas) [18,19]. Contrast administration usually produces intense enhancement, which may extend into the walls of associated polar cysts [20]. Advanced imaging often shows displacement rather than infiltration of white matter tracts on diffusion tensor imaging (DTI) and tractography [9,21] (Table 3).

#### 3.1.2. Spinal Astrocytoma

##### Pilocytic Astrocytoma (Figure 2A)

Pilocytic astrocytomas are the most common intramedullary tumors in children, accounting for 60–70% of pediatric cases, but are rarer in adulthood. Pilocytic astrocytomas most commonly arise in the cervical spinal cord. They are typically eccentric or mildly asymmetric within the spinal cord, and normally well circumscribed, in contrast to diffuse astrocytoma [14]. The longitudinal extent is generally moderate. On MRI, they appear iso- to hypointense on T1- and T2-weighted imaging. Signal homogeneity is common, although mild heterogeneity may be observed due to microcystic changes. Contrast enhancement is variable, but often mild to moderate with patchy, nodular, or faint enhancement [22]. On DTI, they characteristically show reduced fractional anisotropy, with infiltration and disruption of white matter tracts on tractography [23,24]. Perfusion imaging typically demonstrates low to moderately increased relative blood volume compared with high-grade gliomas, reflecting limited neoangiogenesis consistent with their low-grade biological behavior.

**Figure 2:**
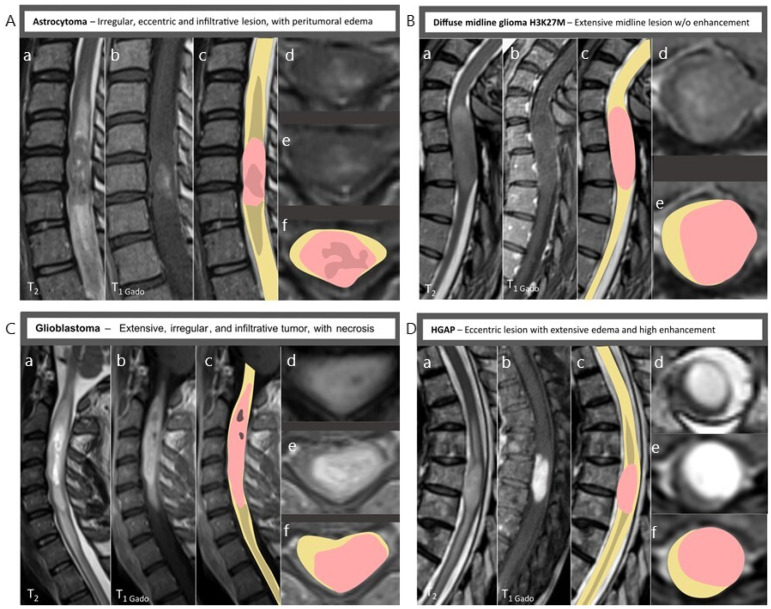
(**A**) **Pilocytic astrocytoma:** (**a**) Sagittal T2-weighted image showing eccentric and irregular intramedullary hyposignal; (**b**) Sagittal T1-contrast-enhanced image showing a patchy enhancement; (**c**) Sagittal pictorial representation; (**d**,**e**) axial T1-contrast-enhanced images showing irregular and patchy enhancement; (**f**) Axial pictorial representation. (**B**) **Diffuse midline glioma H3K27M:** (**a**) Sagittal T2-weighted image showing extensive midline intramedullary mass; (**b**) Sagittal T1-contrast-enhanced image showing the absence of gadolinium enhancement; (**c**) Sagittal pictorial representation; (**d**) Axial T2-weighted image showing a diffuse and eccentric intramedullary mass; (**e**) Axial pictorial representation. (**C**) **Glioblastoma:** (**a**) Sagittal T2-weighted image showing a diffuse hypersignal associated to intramedullary edema; (**b**) Sagittal T1-contrast-enhanced image showing a high enhancement with necrosis; (**c**) Sagittal pictorial representation; (**d**) Axial T2-weighted image; (**e**) Axial T1-contrast-enhanced image showing eccentric lesion; (**f**) Axial pictorial representation. (**D**) **High-grade astrocytoma with piloid features:** (**a**) Sagittal T2-weighted image showing fusiform hyperT2 intramedullary mass with extensive edema; (**b**) Sagittal T1-contrast-enhanced image showing a high enhancement (**c**) Sagittal pictorial representation; (**d**) Axial T2-weighted image; (**e**) Axial T1-contrast-enhanced image; (**f**) Axial pictorial representation.

##### Diffuse Midline Glioma H3K27M (Figure 2B)

Spinal cord diffuse midline glioma, H3K27-altered, most commonly involves the cervical or cervicothoracic levels. These tumors typically present as longitudinally extensive intramedullary lesions with poorly defined margins and marked cord expansion. On MRI, they are usually iso- to hypointense on T1-weighted sequences and heterogeneously hyperintense on T2-weighted images, often extending over multiple vertebral segments. In cases of H3K27-altered diffuse midline gliomas, the enhancement may be surprisingly subtle despite aggressive histology, underscoring the need for integration with advanced imaging markers [25]. Peritumoral edema is common.

##### Glioblastoma (Figure 2C)

Primary spinal cord glioblastoma is exceedingly rare (<1% of all glioblastomas), representing the most aggressive end of the spinal glial tumor spectrum [26]. Glioblastomas can arise anywhere along the spinal cord. They typically present as poorly defined, infiltrative masses causing marked cord expansion with indistinct margins. Longitudinal involvement over multiple vertebral levels is common, and extension into the cervicomedullary junction may occur. Unlike ependymomas, glioblastomas do not produce polar cysts, and syrinx formation is uncommon. On MRI, they typically display a heterogeneous signal on both T1- and T2-weighted sequences, reflecting the coexistence of hypercellular tumor tissue, necrosis, microhemorrhage, and infiltrated normal parenchyma [26]. Contrast administration usually reveals irregular ring-like or patchy enhancement accompanied by substantial perilesional edema [27].

##### High-Grade Astrocytoma with Piloid Features (HGAP) (Figure 2D)

HGAP is a recently characterized infiltrative astrocytic tumor defined by distinct DNA methylation profiling and recurrent MAPK pathway alterations. Spinal HGAP remains rare and may involve the cervical or cervicothoracic cord. On MRI, these tumors typically present as intramedullary expansile lesions with heterogeneous signal characteristics, appearing iso- to hypointense on T1-weighted images and heterogeneously hyperintense on T2-weighted sequences [28]. Unlike classic pilocytic astrocytomas, HGAP frequently demonstrates irregular or heterogeneous contrast enhancement, sometimes with nodular or ring-like components, and may show areas of necrosis or microhemorrhage [28,29]. The margins are often partially infiltrative rather than sharply circumscribed, and longitudinal extension over multiple vertebral segments can occur.

#### 3.1.3. Spinal Glioneuronal Tumor

##### Ganglioglioma (Figure 3A)

Gangliogliomas are low-grade glioneuronal tumors composed of dysplastic ganglion cells mixed with neoplastic glial elements, typically presenting in young adults [30]. Their radiological appearance reflects their mixed cellular composition and slow growth. They typically arise within the thoracic spinal cord and tend to be eccentrically located with well-defined margins. The lesions usually involve one to three vertebral segments, and although they can be expansive, they are less infiltrative than astrocytoma. On MRI, they appear hypointense on T1-weighted images and hyperintense on T2-weighted sequences, but the signal may be heterogeneous due to the presence of microcysts, calcifications, or mixed glioneuronal components. Gangliogliomas usually cause localized cord expansion but are not associated with extensive peritumoral edema, supporting a low-grade process. Their pattern of enhancement is variable, most often mild or patchy rather than intense.

**Figure 3:**
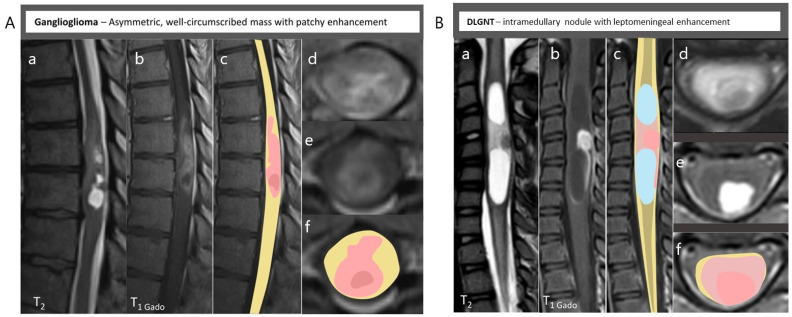
(**A**) **Ganglioma:** (**a**) Sagittal T2-weighted image showing asymmetric intramedullary lesion with intratumoral cysts; (**b**) Sagittal T1-contrast-enhanced image showing a mild enhancement; (**c**) Sagittal pictorial representation; (**d**) Axial T2-weighted image; (**e**) Axial T1-contrast-enhanced image; (**f**) Axial pictorial representation. (**B**) **Diffuse leptomeningeal glioneural tumor:** (**a**) Sagittal T2-weighted image heterogeneous isosignal lesion with hyposignal nodule, associated with syrinx and edema; (**b**) Sagittal T1-contrast-enhanced image showing nodular and subpial enhancement; (**c**) Sagittal pictorial representation; (**d**) Axial T2-weighted image; (**e**) Axial T1-contrast-enhanced image showing a high enhancement of the nodule; (**f**) Axial pictorial representation.

##### Diffuse Leptomeningeal Glioneural Tumors (DLGNTs) (Figure 3B)

DLGNT is a rare glioneuronal neoplasm characterized by diffuse leptomeningeal dissemination. Although primarily affecting children and young adults, spinal involvement is common and may dominate the clinical presentation. On MRI, DLGNT typically manifests as diffuse or nodular leptomeningeal enhancement along the spinal cord, extending over multiple vertebral levels [31]. The underlying spinal cord parenchyma may appear normal or show mild T2 hyperintensity without a discrete intramedullary mass, although secondary cord edema or focal intramedullary nodules can be observed. T1-weighted images are usually unremarkable aside from leptomeningeal thickening, while T2-weighted sequences may demonstrate subtle subpial signal abnormalities [31].

#### 3.1.4. Spinal Hemangioblastoma (Figure 4A)

Hemangioblastomas are benign, highly vascular tumors representing about 3–8% of intramedullary tumors. They may occur sporadically or in the context of von Hippel–Lindau disease. Hemangioblastomas have a predilection for a subpial or exophytic location. They typically present as small, well-circumscribed nodules. On MRI, they appear iso- to hypointense on T1- and T2-weighted sequences, frequently accompanied by a peritumoral syrinx that may extend over several vertebral levels [32]. The classic “cyst-with-nodule” configuration is highly suggestive, with the enhancing mural nodule corresponding to the solid, vascular component of the tumor [32,33]. After contrast administration, they characteristically show intense nodular enhancement, and feeding or draining vessels are commonly visualized as serpiginous flow voids on T2 or SWI sequences. Spinal angiography allows the delineation of arterial feeders and venous drainage, confirming the diagnosis and aiding operative strategy [32].

**Figure 4:**
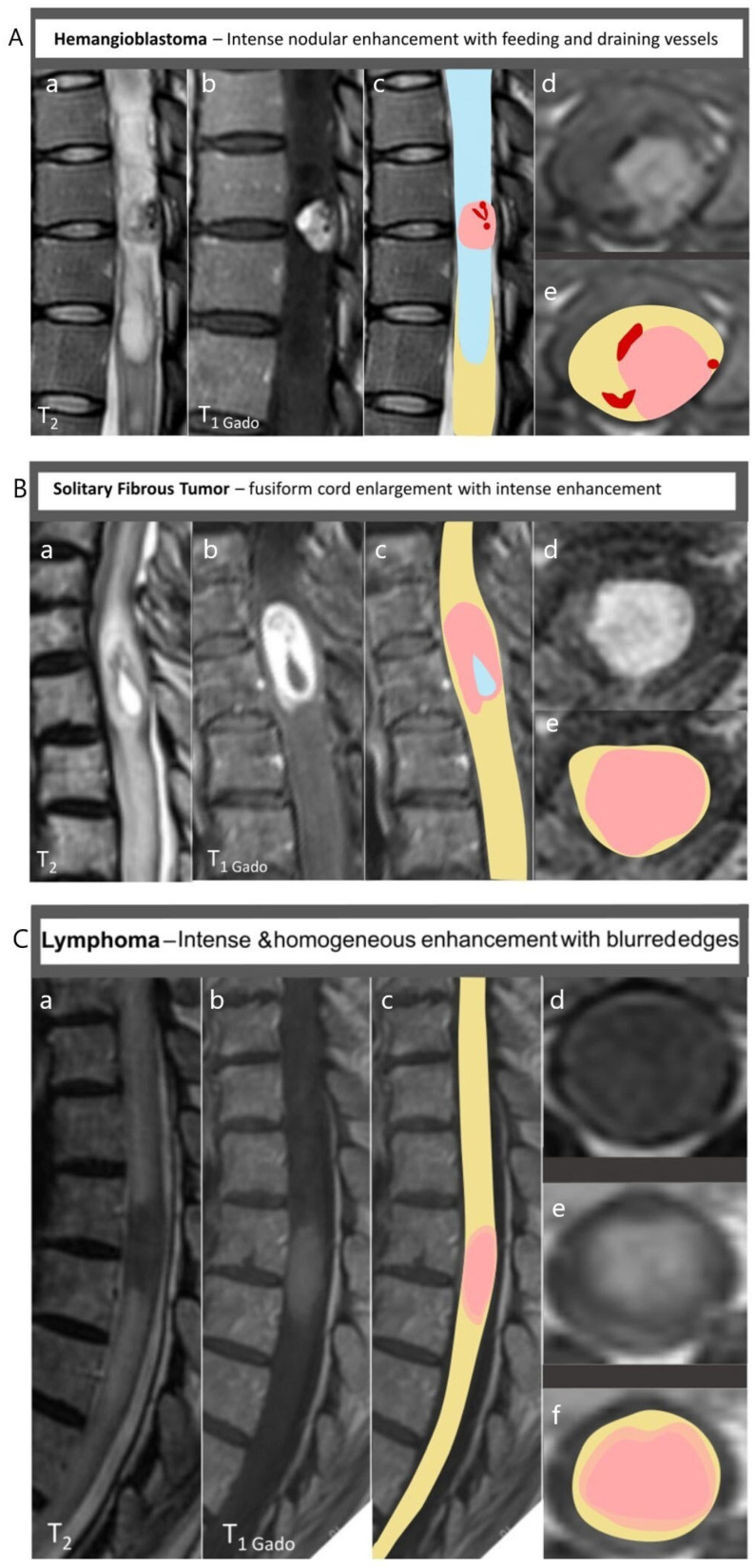
(**A**) **Hemangioblastoma:** (**a**) Sagittal T2-weighted image showing a hyposignal nodule with draining vessels associated to syrinx; (**b**) Sagittal T1-contrast-enhanced image showing an intense enhancement; (**c**) Sagittal pictorial representation; (**d**) Axial T1-contrast-enhanced image showing a nodular tumor with hyposignal draining vessels at the ventral and dorsal poles of the tumor; (**e**) Axial pictorial representation. (**B**) **Solitary fibrous tumor**: (**a**) Sagittal T2-weighted image showing a fusiform intramedullary tumor with extensive edema; (**b**) Sagittal T1-contrast-enhanced image showing intense enhancement; (**c**) Sagittal pictorial representation; (**d**) Axial T1-contrast-enhanced image; (**e**) Axial pictorial representation. (**C**) **Lymphoma:** (**a**) Sagittal T2-weighted image showing intramedullary hyposignal; (**b**) Sagittal T1-contrast-enhanced image showing homogeneous enhancement with blurred edges; (**c**) Sagittal pictorial representation; (**d**) Axial T2-weighted image; (**e**) Axial T1-contrast-enhanced image; (**f**) Axial pictorial representation.

#### 3.1.5. Solitary Fibrous Tumor (Figure 4B)

Hemangiopericytomas, now classified within the solitary fibrous tumor (SFT) spectrum, are rare but clinically important due to their recurrence potential. SFTs can be intradural extramedullary, intra-extramedullary, or intramedullary only [34,35]. Intramedullary SFTs usually appear as well-demarcated, expansile masses causing fusiform cord enlargement. On MRI, the solid component is iso- to hypointense on T1-weighted images and commonly hypointense on T2-weighted sequences, a feature attributed to their dense collagen content [36]. Areas of intermediate or slightly hyperintense T2 signal may appear when cellularity increases. After contrast administration, they demonstrate intense and homogeneous enhancement with sharp margins, and present without associated polar cysts, helping differentiate them from ependymomas [34,36].

#### 3.1.6. Lymphoma (Figure 4C)

Primary intramedullary lymphoma is uncommon (most often arising in the context of primary CNS lymphoma or systemic lymphoproliferative disease), but should be included in the differential diagnosis of enhancing intramedullary lesions. Intramedullary lymphomas typically present as diffuse or fusiform cord enlargement rather than a discrete mass [33,37]. On MRI, lymphoma typically appears iso- to hypointense on T1-weighted sequences and hypointense on T2-weighted images, often accompanied by diffuse swelling of the spinal cord [33,37]. Following gadolinium administration, it demonstrates intense and homogeneous enhancement [37], a feature that reflects its dense cellular architecture rather than true hypervascularity. Moreover, tumor contours may be blurred after gadolinium injection. Diffusion-weighted imaging (DWI) usually shows marked diffusion restriction with low apparent diffusion coefficient (ADC) values, consistent with the high cellularity characteristic of lymphoma. Perfusion imaging shows low rCBV despite marked enhancement, differentiating it from hypervascular gliomas.

### 3.2. Metastases

#### 3.2.1. Secondary CNS Tumors: Metastases (Figure 5A)

Intramedullary metastases are a very rare intramedullary entity, most frequently from lung, breast, renal cell carcinoma or melanoma primaries [38,39]. They frequently involve the cervical or lower thoracic spinal cord, consistent with the regional vascular supply and higher blood flow in these segments. Metastases often involve shorter cord segments than diffuse gliomas, but multiple lesions may be present. On MRI, intramedullary metastases are often eccentric or subpial in location [39]. They appear iso- to hypointense on T1-weighted images while hyperintense on T2-weighted sequences is usually observed due to extensive peritumoral vasogenic edema [39]. Gadolinium enhancement is typically intense, nodular, reflecting breakdown of the blood–spinal cord barrier [39]. After contrast injection, the “flame sign” is characteristic (corresponding to a leptomeningeal involvement), reflecting dissemination along the subarachnoid space.

**Figure 5:**
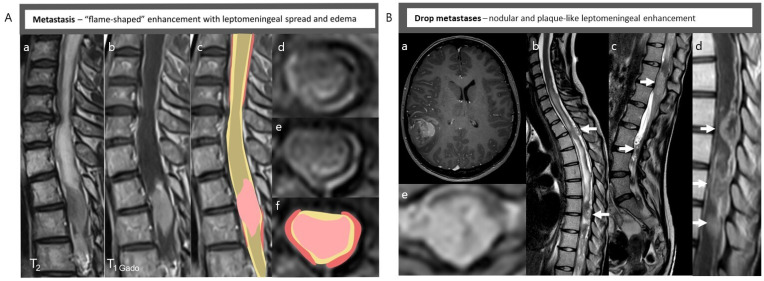
(**A**) **Metastases:** (**a**) Sagittal T2-weighted image showing a hyposignal intramedullary mass with extensive edema; (**b**) Sagittal T1-contrast-enhanced image showing an intense enhancement with leptomeningeal dissemination; (**c**) Sagittal pictorial representation; (**d**,**e**) Axial T1-contrast-enhanced image; (**f**) Axial pictorial representation. (**B**) **Drop metastases:** (**a**) Axial T1-contrast-enhanced image showing a posterior temporal glioblastoma; (**b**,**c**) Sagittal T2-weighted images of the entire spinal cord showing multiple (arrows) extraxial and intraxial nodule, with extensive intramedullary edema; (**d**) Sagittal T1-contrast-enhanced image; (**e**) Axial T1-contrast-enhanced image showing extra/intra axial tumor.

#### 3.2.2. Drop Metastases (Figure 5B)

In addition, secondary spinal involvement may also occur through cerebrospinal fluid dissemination, commonly referred to as “drop metastases.” These lesions result from tumor cell spread along the neuraxis from an intracranial primary tumor, most classically medulloblastoma, intracranial ependymoma [40], or high-grade gliomas [41]. Spinal drop metastases typically present as nodular or plaque-like leptomeningeal enhancement along the spinal cord or cauda equina rather than as true parenchymal intramedullary masses [42]. Although less common, intramedullary implantation has been described in this context. Recognition of this dissemination pathway is essential, as it significantly alters staging, prognosis, and therapeutic strategy, necessitating complete craniospinal imaging.

### 3.3. Non-Neoplastic Intramedullary Lesions

#### 3.3.1. Cavernoma (Figure 6A)

Cavernous malformations are composed of dilated capillary channels without intervening neural tissue. Although they are non-neoplastic lesions, they are an important diagnostic consideration in the differential diagnosis of intramedullary tumors due to their mass effect and hemorrhagic potential. Intramedullary cavernomas can occur at any spinal level. They typically present as well-defined, compact lesions, and are eccentric and/or exophytic [43,44]. On MRI, they typically show a mixture of hyperintense and hypointense signal on both T1- and T2-weighted sequences, producing the classic “popcorn” or “mulberry”-like appearance that reflects blood products of varying ages [43,45]. On susceptibility-weighted imaging, they are surrounded by a prominent hypointense rim caused by hemosiderin deposition. Contrast enhancement is generally absent, helping distinguish them from true neoplastic lesions [43,45].

**Figure 6:**
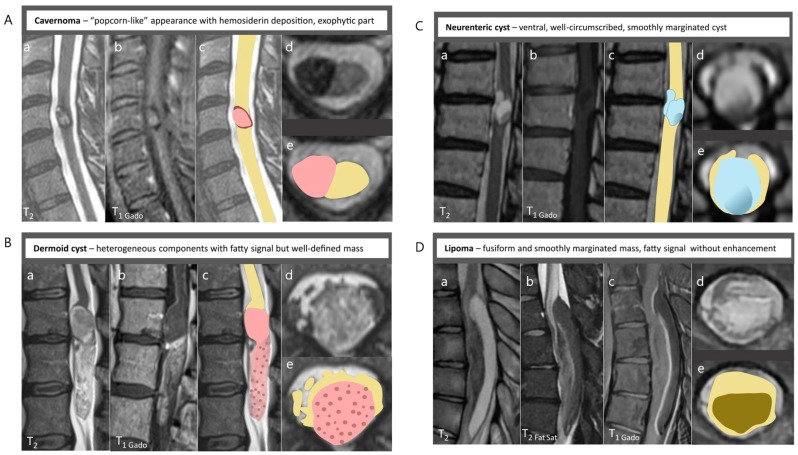
(**A**) **Cavernoma:** (**a**) Sagittal T2-weighted image showing “popcorn-like” appearance hemosiderin deposition; (**b**) Sagittal T1-contrast-enhanced image showing the absence of gadolinium enhancement; (**c**) Sagittal pictorial representation; (**d**) Axial T2-weighted image showing exophytic part of the cavernoma within the cord; (**e**) Axial pictorial representation. (**B**) **Dermoid cyst:** (**a**) Sagittal T2-weighted image showing a well-defined mass within the conus medullaris; (**b**) Sagittal T1-contrast-enhanced image showing the absence of gadolinium enhancement; (**c**) Sagittal pictorial representation; (**d**) Axial T2-weighted image showing the heterogeneous component of the tumor; (**e**) Axial pictorial representation. (**C**) **Neurenteric cyst:** (**a**) Sagittal T2-weighted image showing a well-circumscribed bilobed cyst; (**b**) Sagittal T1-contrast-enhanced image showing the absence of gadolinium enhancement; (**c**) Sagittal pictorial representation; (**d**) Axial T2-weighted image showing an intramedullary cyst opening the spinal cord ventrally, with CSF flux inside; (**e**) Axial pictorial representation. (**D**) **Lipoma:** (**a**) Sagittal T2-weighted image showing fusiform hyper-T2 mass within the dorsal columns; (**b**) Sagittal T2 FatSat-weighted image showing a hyposignal (**c**) Sagittal T1-contrast-enhanced image showing the absence of gadolinium enhancement; (**d**) Axial T2-weighted image showing the intramedullary counterpart of the lipoma at the cranial pole; (**e**) Axial pictorial representation.

#### 3.3.2. Dermoid and Epidermoid Cysts (Figure 6B)

Dermoid and epidermoid cysts of the spinal cord are rare congenital lesions arising from ectodermal inclusions during neural tube closure. They are most frequently encountered in the lumbosacral region and in association with spinal dysraphism [46]. When intramedullary, they present as well-defined, slowly expanding lesions that may cause focal cord enlargement. Dermoid cysts tend to be more heterogeneous due to their complex contents, whereas epidermoid cysts are typically more uniform. Dermoid cysts, which contain fat, hair, and sebaceous material, often appear hyperintense on T1-weighted sequences and heterogeneously hyperintense on T2 [46,47], with signal characteristics influenced by lipid-rich components. Epidermoid cysts, composed of keratin debris, usually demonstrate T1 hypointensity or isointensity and T2 hyperintensity, often resembling CSF but with slightly higher signal intensity [47,48]. Neither dermoid nor epidermoid cysts typically exhibit contrast enhancement. Thin peripheral enhancement may occasionally be observed in dermoid cysts if an inflammatory reaction is present, but the cyst contents themselves do not enhance.

#### 3.3.3. Neurenteric Cysts (Figure 6C)

Neurenteric cysts are benign congenital lesions resulting from abnormal notochord–foregut separation during embryologic development [49]. Although they are rare, when intramedullary, they can mimic a spinal cord tumor. On MRI, they are typically located ventral to the spinal cord [49], most commonly in the cervical or upper thoracic region. Morphologically, these cysts are well circumscribed and non-expansile, usually causing only mild compression of the adjacent spinal cord without infiltrative features [48,49]. Their signal characteristics reflect their proteinaceous or mucinous content, appearing hyperintense on both T1- and T2-weighted sequences [48,49], and they do not enhance after gadolinium administration, reflecting the absence of a vascularized solid component, consistent with their benign epithelial nature. Diffusion imaging shows no restriction, confirming a cystic rather than solid nature. In rare cases, however, susceptibility-weighted imaging (SWI) has demonstrated a calcified cystic component within an intramedullary neurenteric cyst, appearing as marked susceptibility signal drop-out [50].

#### 3.3.4. Lipomas (Figure 6D)

Intramedullary lipomas are rare congenital lesions, and can be associated with dysraphic anomalies such as spinal dysraphism or diastematomyelia. They result from abnormal inclusion of mesenchymal tissue during neural tube closure rather than representing true neoplasms. Intramedullary lipomas typically occur in the thoracic cord and often present as fusiform, smoothly marginated masses expanding the spinal cord [51,52]. On MRI, they show a characteristic fat-equivalent signal pattern: the lesion is hyperintense on T1-weighted sequences and hyperintense on T2, mirroring the signal of subcutaneous fat [51,52]. Fat-suppressed sequences or STIR imaging produce a marked signal drop, confirming the fatty nature of the lesion [51,52]. Contrast enhancement is absent, although thin septations may occasionally display minimal enhancement.

## 4. Advances in Imaging Biomarkers

The growing availability of multiparametric MRI techniques has expanded the diagnostic scope of spinal cord imaging beyond morphology, allowing the characterization of tumor microstructure, vascularity, and metabolism.

### 4.1. DWI

**Figure 7:**
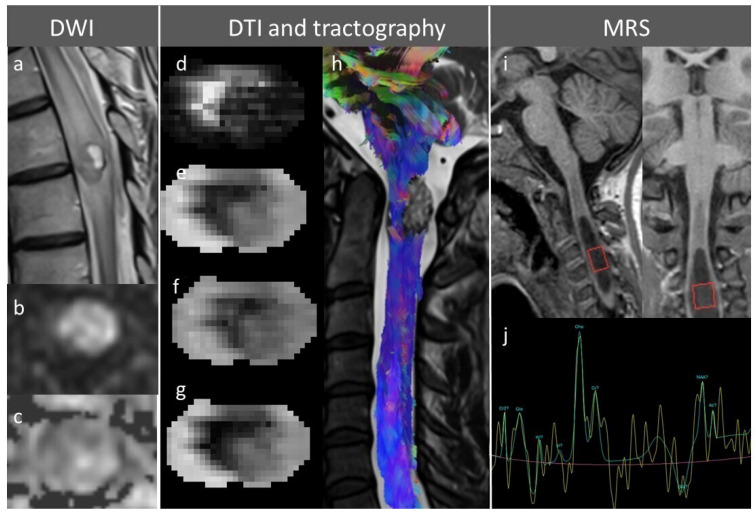
(**a**) Sagittal T2-weighted image showing intramedullary tumor with extensive edema; (**b**) Axial diffusion weighted-image of the intramedullary tumor presented in panel (**a**) showing hypersignal; (**c**) Apparent diffusion coefficient map of the intramedullary tumor presented in panel (**a**) showing hypersignal; (**d**) Fractional anisotropy (FA) map of cervical spinal cord with cavernoma showing decreased FA (i.e., microarchitectural disorganization); (**e**) Mean diffusivity (MD) map of cervical spinal cord with cavernoma showing increase MD; (**f**) Axial diffusivity map of cervical spinal cord with cavernoma; (**g**) Radial diffusivity map of cervical spinal cord with cavernoma; (**h**) Spinal cord tractography showing displacement of spinal white matter due to cervical cavernoma; (**i**) Sagittal and coronal views showing region of interest placement for spectroscopy in case of cervical diffuse leptomeningeal glioneural tumor; (**j**) Spectroscopy of diffuse leptomeningeal glioneural tumor showing a high pic of choline and creatine, demonstrating increased membrane turnover and tumor proliferation.

DWI provides a rapid, quantitative assessment of tumor cellularity through measurement of the ADC [53]. Restricted diffusion (low ADC) is caused by decreased free motion of water molecules and characterizes hypercellular tumors (such as lymphoma) [53] or high protein content (such as in epidermoid cysts) [54]; facilitated diffusion (high ADC) is observed in low-grade astrocytomas (Figure 7).

### 4.2. DTI and Tractography

DTI quantifies the anisotropy of water diffusion within white-matter tracts, providing insights into the organization and integrity of spinal cord fibers [55]. It is particularly useful for distinguishing displacing from infiltrating lesions: ependymomas tend to displace spinal tracts, leading to sharply demarcated margins on color-coded fractional anisotropy (FA) maps [6,21], whereas pilocytic astrocytomas and high-grade gliomas typically infiltrate surrounding fibers, resulting in decreased FA and elevated mean diffusivity due to the disruption of tract coherence (often associated with a decrease in axial diffusivity corresponding to axonal loss and an increase in radial diffusivity corresponding to the demyelination process) [23]. Clinically, DTI-based tractography assists neurosurgeons in differentiating spinal tracts from one another and providing preoperative planning that defines the relationship of each tract of interest to the intramedullary tumor, finally establishing predictive factors for tumor resectability [6,7,56] (Figure 7).

### 4.3. Magnetic Resonance Spectroscopy (MRS)

MRS provides metabolic fingerprints by quantifying resonances from choline (Cho, biomarker of cellular turn over), N-acetylaspartate (NAA, biomarker of neural viability), creatine (Cr, biomarker of energy metabolism), lactate (biomarker of anaerobic metabolism), and lipids, which can help to determine tumor grade, biological activity, and response to therapy [57]. Semi-quantitative analysis using ratios of various metabolites is used to better predict the grade. Intramedullary ependymomas show strongly reduced NAA/Cr levels, whereas Cho/Cr and myoInosotol/Cr are moderately increased [10]. In contrast, high-grade intramedullary tumors tend to present a decreased NAA and Cr, with a high peak of myo-inositol (a biomarker of astrocyte proliferation) [58]. These metabolic patterns complement morphological findings and may provide indirect information on tumor aggressiveness and biological behavior. However, technical limitations—including voxel size constraints, partial volume effects, low signal-to-noise ratio, and susceptibility to motion—currently restrict the routine use of spinal cord MRS, confining its role mainly to specialized centers and research settings [57] (Figure 7).

### 4.4. Perfusion-Weighted Imaging (PWI)

PWI, using dynamic susceptibility contrast or dynamic contrast-enhanced techniques, allows the quantitative assessment of tumor vascularity through parameters such as relative blood volume (rCBV). In intramedullary tumors, PWI enables vascular phenotyping, which complements conventional contrast-enhanced MRI. High-grade gliomas [11,59] and glioblastomas [60] consistently demonstrate markedly elevated rCBV, correlating with dense capillary networks and microvascular proliferation. However, clinical adoption remains limited by technical challenges related to the small cord diameter, motion and CSF-pulsation artifacts, and the lack of standardized acquisition and post-processing protocols [59].

## 5. Discussion

Intramedullary tumors represent a rare but clinically critical subset of CNS neoplasms [1,61]. Despite their histological diversity, a structured analysis of imaging features—integrating morphology, signal characteristics, and enhancement patterns—can substantially narrow differential diagnosis and guide optimal management. Today, conventional MRI remains the cornerstone of evaluation, providing essential information on lesion localization, longitudinal extent, and cord morphology [5,62]. Nevertheless, advanced MRI techniques could revolutionize diagnostic accuracy by defining radiological biomarkers specific to each histological entity.

This pictorial review provides a structured overview of MRI features across a broad spectrum of histologically confirmed intramedullary lesions. Across the entities illustrated in this study—ranging from glial tumors (subependymoma, ependymoma, pilocytic astrocytoma, high-grade astrocytoma, glioblastoma, oligodendroglioma, ganglioglioma) to secondary neoplasms (metastases, lymphoma), vascular lesions (hemangioblastoma, cavernoma), mesenchymal tumors (solitary fibrous tumor), and developmental lesions (epidermoid cyst, neurenteric cyst, lipoma)—distinct imaging phenotypes emerge. These phenotypes are shaped by fundamental biological traits: expansile versus infiltrative growth, cellular density, vascular proliferation, cyst formation, hemorrhagic propensity, and preservation or disruption of the blood–spinal cord barrier. This concept aligns with the broader evolution of neuro-oncology toward biologically informed classification systems [12] and supports the idea that radiological assessment, when structured and systematic, may contribute to a more mechanistic understanding of spinal cord disease.

**Figure 8:**
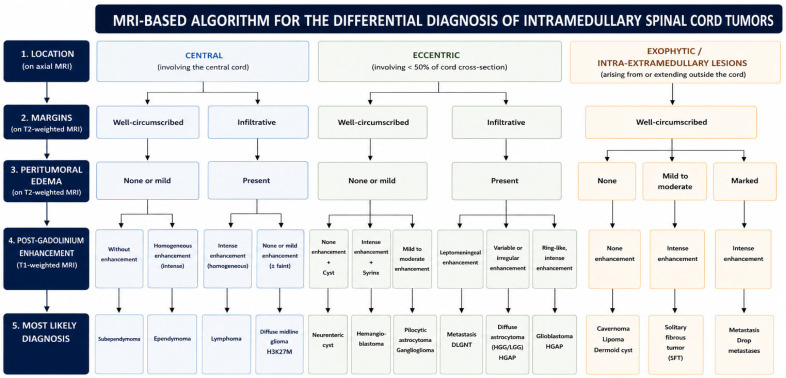
MRI-based algorithm for the differential diagnosis of intramedullary spinal cord tumors.

The imaging features described in our study are consistent with prior reports emphasizing the importance of lesion location, morphology, and enhancement patterns in narrowing the differential diagnosis [14,17,20,25,32]. Beyond confirming previously described imaging patterns, the present work adopts a didactic, pattern-based approach designed to reflect real-world diagnostic reasoning. In daily clinical practice, radiological interpretation relies less on isolated imaging signs than on a stepwise integration of key features, including lesion location, margin definition, enhancement pattern, and the presence of peritumoral edema or associated findings (Figure 8).

The clinical value of this framework lies in its ability to inform decision-making in a context where biopsy or extensive resection carries significant neurological risk [63]. In intramedullary pathology, surgical strategy is closely tied to anticipated tumor behavior. Lesions with imaging features suggesting expansile growth (e.g., ependymoma, hemangioblastoma) are often approached with an intent of maximal safe resection [63,64,65], whereas imaging patterns consistent with infiltrative gliomas or lymphoma may favor limited resection, biopsy, or primarily oncological treatment. Therefore, structured radiological interpretation functions not only as a diagnostic exercise but also as a form of risk stratification and therapeutic guidance.

### 5.1. Future Directions

The future of intramedullary tumor imaging lies in moving beyond qualitative morphologic assessment toward quantitative, biologically informative imaging biomarkers. While conventional MRI provides essential structural information, it remains predominantly descriptive and insufficient to fully capture the microstructural, vascular, and metabolic heterogeneity that defines tumor behavior. A multiparametric approach, integrating advanced MRI techniques, will offer a pathway toward radio-genomics or radio-molecular correlations.

As the 2021 WHO classification [12] increasingly integrates genomic and epigenetic features into tumor taxonomy, imaging must evolve toward a radio-molecular paradigm, where MRI serves as a proxy for underlying molecular identity. The emerging field of radio-genomics will seek to establish robust correlations between quantitative imaging metrics and genomic alterations, enabling early and non-invasive molecular profiling. Such signatures could support growth pattern, or biological aggressiveness, thereby contributing to risk stratification and personalized treatment planning, aligning diagnostic radiology with the molecular era of neuro-oncology.

Ultra-high-field MRI (7T) further expands this perspective. Its improved signal-to-noise ratio and spatial resolution may enhance visualization of gray–white matter interfaces [66], microvascular architecture (due to enhancement of susceptibility-based contrast), and subtle infiltrative changes, potentially refining margin delineation and tract involvement. In this perspective, 7T MRI does not merely represent a higher-resolution version of conventional imaging but rather a shift toward microstructural and vascular sensitivity that may better capture the biological footprint of intramedullary tumors. Its integration with multiparametric analysis and computational modeling could contribute to establishing imaging-derived biomarkers that more closely reflect tumor infiltration and microenvironmental alterations.

### 5.2. Strengths and Limitations

A key strength of this study is the use of histopathologically confirmed cases as a reference standard, ensuring that the illustrated imaging patterns are grounded in definitive diagnoses according to current WHO classification criteria. The structured, entity-based organization reflects real-world clinical reasoning and encompasses both common and rare intramedullary lesions, thereby providing a didactic and practical framework. However, several limitations should be acknowledged. First, the retrospective design introduces inherent selection bias, since MRI acquisition protocols were not fully standardized and have evolved over time. Second, this is a single-center study, which may limit the generalizability of the findings. In addition, for each tumor entity, only a single representative case was selected; therefore, the spectrum of imaging variability within a given histological subtype is not fully captured, and atypical presentations may not be reflected. Moreover, the relatively limited sample size for rare tumor subtypes restricts the generalizability of specific imaging patterns. While advanced MRI techniques, including DTI, perfusion imaging, and MRS, have demonstrated potential in improving lesion characterization and providing insights into tumor microstructure and vascularity, their clinical use in spinal cord imaging remains limited by technical constraints, susceptibility artifacts, and the lack of standardized acquisition [67,68]. Furthermore, the analysis is based exclusively on MRI, without integration of other imaging modalities. Finally, significant overlap persists between the imaging features of different tumor entities, and radiological phenotypes, while highly informative, cannot replace histopathological and molecular diagnosis, which remain the gold standard.

## 6. Conclusions

Intramedullary tumors represent a diagnostically and therapeutically challenging group of spinal cord lesions in which clinical decision-making must balance oncological control with preservation of neurological function. This review highlights that a structured MRI approach can help differentiate major tumor entities and guide preoperative strategy.

Beyond conventional morphological assessment, emerging multiparametric and ultra-high-field MRI techniques provide additional insights into tumor microstructure, vascularity, and metabolism. Although currently limited by technical and standardization constraints, these approaches hold promise for the development of non-invasive imaging biomarkers and for strengthening radiological–molecular correlations. In this evolving paradigm, MRI is expected to play an increasingly central role not only in diagnosis but also in treatment planning and radiogenomic characterization of intramedullary spinal cord tumors.

## Figures and Tables

**Table 1:** Pathological classification of intramedullary lesions according to the 2021 WHO classification system.

Intramedullary Tumor Histologies			Genetic Alterations	Key MRI Features
**Primitive CNS tumors**				
	**Spinal ependymal tumors**	Spinal subependymoma	Unknown	Non-enhancing, well circumscribed
		Spinal ependymoma	NF2 mutation or deletion	Central, homogeneous enhancement, polar cysts, intratumoral cysts
		Spinal ependymoma MYCN	MYCN amplification	
	**Spinal astrocytoma**	Pilocytic astrocytoma	MAPK pathway alterations, especially BRAF V600E mutation, KIAA1549-BRAF fusion	Irregular, eccentric, well defined, mild enhancement
		Diffuse astrocytoma	IDH mutation (rare), ATRX loss, TP53 mutation, BRAF V600E mutation	Infiltrative, long segment
		Spinal glioblastoma IDH wild-type	EGFR amplification, TERT promoter mutation, TP53	Extensive, irregular, necrosis, ring enhancement
		Diffuse midline glioma H3K27M altered	H3K27, EGFR alteration, TP53, ATRX mutation	Extensive, poorly defined, mild enhancement
		High-grade astrocytoma with piloid feature	MAPK pathway, CDKN2A/B deletion	Eccentric with extensive edema and high enhancement
	**Spinal glioneuronal tumor**	Gangliogliomas	BRAF V600E mutation	Asymmetric, well-defined, mild enhancement
		Diffuse leptomeningeal glioneural tumor	KIAA1549-BRAF fusion, 1p/19q codeletion, IDHwt	Diffuse leptomeningeal enhancement
	**Spinal hemangioblastoma**		VHL	Cyst with mural nodule, flow voids
	**Spinal cord solitary fibrous tumor**		NAB2–STAT6 fusion	Well-defined, intense enhancement
	**Spinal cord lymphoma**		MYD88, CD79B	intense and homogeneous enhancement
**Spinal cord Metastases**				
	**Secondary CNS tumors: metastases**		Variable	Nodular/ring, edema, flame sign
	**Drop metastases**		CSF dissemination	Leptomeningeal nodules
**Non-neoplastic tumors**				
	**Spinal cavernoma**		KRIT1, CCM genes	“Popcorn” appearance, no enhancement
	**Dermoid cyst**		Developmental	Fat signal
	**Epidermoid cyst**		Developmental	CSF-like signal
	**Neurenteric cyst**		Developmental	Ventral, well-circumscribed, smooth-margined cysts
	**Lipoma**		Developmental	Fusiform fat signal

**Table 2:** MRI protocol.

Parameters	Specification
**Field strength**	3T for higher spatial resolution
**Coil**	Dedicated spine coil
**Slice thickness**	≤3 mm (no gap if possible)
**Sequences**	**Purpose**
**T1-weighted pre-contrast (sagittal ± axial)**	Baseline anatomy, detection of hemorrhage, fat, and intrinsic signal characteristics
**T2-weighted** **(sagittal + axial)**	Lesion detection, cord expansion, edema, cysts, syrinx
**T1-weighted post-gadolinium** **(sagittal + axial)**	Assessment of blood–spinal cord barrier disruption and enhancement pattern

**Table 3:** Radiological comparison of ependymoma and astrocytoma.

Feature	Ependymoma	Astrocytoma (Diffuse/High-Grade)
**Location (axial)**	Central, symmetric	Eccentric, asymmetric
**Margins**	Well circumscribed	Poorly defined, infiltrative
**Cord expansion**	Symmetric fusiform enlargement	Asymmetric, often irregular
**T1 signal**	Iso–hypointense	Iso–hypointense
**T2 signal**	Homogeneous hyperintensity	Heterogeneous, often with edema
**Enhancement pattern**	Intense, homogeneous	Variable; patchy, heterogeneous
**Polar cysts**	Frequent	Rare
**Intratumoral cysts**	Frequent	Common
**Hemorrhage/“cap sign”**	Characteristic (hemosiderin rim)	Rare
**Edema**	Limited	Frequent and extensive
**DTI (tractography)**	Displacement of fibers	Infiltration and disruption
**Surgical implication**	Favorable cleavage plane	Difficult resection (no clear plane)

## Data Availability

The data presented in this study are available on request from the corresponding author due to ethical restrictions.
